# Vitamin D deficiency and thyroid cancer: is there a true association? A prospective observational study

**DOI:** 10.1308/rcsann.2024.0041

**Published:** 2024-09-24

**Authors:** S Lanitis, V Gkanis, S Peristeraki, P Chortis, N Kalogeris, A Vryonidou

**Affiliations:** ^1^ 2nd Surgical Department and Unit of Surgical Oncology, ‘Korgialeneio â Benakeio’, Hellenic Red Cross, General Hospital of Athens, Greece; ^2^ Endocrinology Department, ‘Korgialeneio – Benakeio’, Hellenic Red Cross, General Hospital of Athens, Greece

**Keywords:** Vitamin D, Thyroid cancer, Thyroid surgery

## Abstract

**Introduction:**

Literature data indicate a correlation between vitamin D deficiency and thyroid cancer (TC). We conducted this observational study to test this hypothesis.

**Methods:**

We studied 327 consecutive thyroidectomy cases, and compared patients with TC and those who had benign thyroid disease (BTD). In total, 183 cases with well-differentiated TC (group B) were compared with 144 cases of BTD (group A). We defined 25-hydroxyvitamin D (25(OH)VitD) values <10ng/ml as severe vitamin D deficiency (15.4%), 10–30ng/ml as inadequacy (70.4%) and >30ng/ml as adequate (14.2%). We further used a cut-off point of 30ng/ml (used in a recent meta-analysis) to classify patients as vitamin D deficient or not.

**Results:**

There was no statistically significant difference in the following: age, size of the thyroid gland, preoperative calcium levels, preoperative parathormone and vitamin D levels, body mass index and anti-thyroid antibodies. Only thyroid-stimulating hormone and weight of the thyroid gland were found to differ. There was no significant difference in mean vitamin D levels (group A = 19.82ng/ml [sd 9.59] vs group B = 19.69ng/ml [sd 11.34]; *p* = 0.917). The same was found when we compared the two groups according to the three categories of vitamin D values (deficiency, inadequacy, adequacy; *p* = 0.485) and when we performed the analysis based on all threshold levels (10, 20 and 30ng/ml; *p* = 0.328). Using various statistical methods, no correlation was found between vitamin D deficiency and differentiated TC (overall, microcarcinomas, macrocarcinomas).

**Conclusions:**

Based on our results, no correlation between vitamin D deficiency and TC was confirmed, contradicting and questioning the results of two recent meta-analyses.

## Introduction

Thyroid cancer (TC) is the most common endocrine malignancy with an increasing incidence over the past 30 years, most probably because of overdiagnosis.^1^ Recently, two meta-analyses investigated the correlation of vitamin D deficiency and TC, and both found an association of low 25-hydroxyvitamin D (25(OH)VitD) levels with TC.^2,3^ The first meta-analysis concluded that vitamin D deficiency may be a risk factor for TC, whereas the other came to more or less the same conclusion, by finding that high levels of circulating vitamin D decrease the risk for TC. ^2,3^ In addition to its well-known role in bone metabolism and regulating calcium homeostasis, vitamin D is known to regulate a large number of genes.^4^ It has been suggested that there is an association between vitamin D deficiency and chronic diseases, including cancer.^4^ Analysis of a National Health Service cohort (*n* = 32,826) revealed an inverse correlation between 25(OH)VitD levels and risk for colorectal cancer, a finding also reported in meta-analyses for the 2008 International Agency for Research on Cancer report. Similar findings emerge from other meta-analyses, demonstrating a protective effect of vitamin D on breast cancer.^5,6^ However, despite a possible correlation between vitamin D deficiency and various types of cancer, when it comes to TC, any correlation remains unclear. Moreover, low levels of vitamin D have been associated with autoimmune thyroid diseases (Hashimoto's thyroiditis and Graves' disease).^7,8^ Although some research failed to prove so, several other studies, including a meta-analysis, have shown that people with these conditions have low levels of vitamin D, suggesting an association, at least for certain group of patients.^8^ 25(OH)VitD (calcidiol) accounts for the majority of circulating and stored forms of vitamin D and hence represents the best marker with which to assess total body vitamin D levels.^9^ There is debate over the definition of vitamin D deficiency. Levels below 20ng/ml are considered as deficiency and levels between 20 and 30ng/ml as insufficiency.^7^ Other authors use 10ng/ml as the point at which they distinguish deficiency from insufficiency.^7^

In terms of an association between vitamin D deficiency and cancer there are inconsistent data in the literature and, although epidemiological studies have supported the association of the protective effect of vitamin D, no randomised controlled studies have confirmed this.^7^ The same is true for TC. Some studies suggest that vitamin D deficiency may increase the incidence of TC and even lead to worse outcomes in these patients, whereas other studies have failed to show any association.^7^ The purpose of this study was to assess whether there is indeed an association between low vitamin D levels and TC, and whether there is a clear correlation of different thyroid pathology with this condition.

## Methods

The study was approved by the ethics committee of the ‘Korgialeneio – Benakeio’, Hellenic Red Cross, General Hospital of Athens. To conduct the study, we included all patients who underwent thyroidectomy, both for benign conditions and well-differentiated thyroid carcinomas, during the study period. Patients who underwent surgery for medullary carcinomas, those with parathyroid gland disorders and patients taking oral vitamin D supplements, were excluded. Written consent was obtained from each patient, after full explanation of the purpose and nature of all the procedures used. We maintain a prospectively collected database, and vitamin D has been recorded regularly during the past 3 years for all patients undergoing surgery. Overall, 327 consecutive patients were enrolled between January 2017 and January 2020. All patients were referred to our centre by endocrinologists from all over the Greek territory, as well as from the endocrinology department of our hospital, ensuring no selection bias. We measured 25(OH)VitD levels, calcium levels and parathormone (PTH) levels just before surgery for all patients, along with the basic blood tests and thyroid function tests (thyroid-stimulating hormone [TSH], triiodothyronine [T3], free-thyroxine [FT4], thyroglobulin [Tg], thyroid peroxidase antibodies [anti-TPO], thyroglobulin antibodies [anti-Tg], calcitonin). All patients had undergone the appropriate imaging tests according to their thyroid pathology. In 183 of 327 patients, well-differentiated TC was found in the final histological report (group B), whereas in 144 cases benign thyroid disease (BTD) was confirmed (group A). The study was conducted in Greece, where there is more than adequate sun exposure, and patients were assessed throughout the year covering all seasons and reflecting the overall vitamin D status of the population. By contrast to other studies, the control group consisted of patients with underlying thyroid pathology to exclude that as a confounding factor.

### Vitamin D levels

There are variations in the literature concerning the normal range for 25(OH)VitD levels. Values below 10ng/ml are considered severely deficient by every standard, including the Institute of Medicine, the Endocrine Society and the Vitamin D Council. Levels between 10 and 20ng/ml are considered inadequate by Endocrine Society and Vitamin D Council standards, whereas the Institute of Medicine considers them mildly deficient or insufficient. Levels between 20 and 30ng/ml are considered deficient by the Vitamin D Council, whereas according to the Endocrine Society, these levels are insufficient; by the Institute of Medicine's standards, these levels are adequate. Levels over 30ng/ml are considered sufficient by the Endocrine Society and Institute of Medicine, but still insufficient by the Vitamin D Council. Finally, by all standards, levels above 40ng/ml are considered sufficient.

In Greece we mainly use the Endocrine Society standards for the classification of vitamin D adequacy. Therefore, we initially classified our patients as severely deficient (<10ng/ml), inadequate (10–30ng/ml) and adequate (>30ng/ml). Most patients (70.4%) had inadequate levels of vitamin D (10–30ng/ml). Severe insufficiency was recorded in 15.4%, whereas only 47 patients (14.2%) had sufficient levels (>30ng/ml). For the purpose of the study, we also made three other classifications according to the insufficient vs sufficient limit: universally accepted (<10ng/ml), mostly accepted (<20ng/ml) and Vitamin D Council accepted (<30ng/ml). Using the 10ng/ml threshold, 15.4% of patients were classified as vitamin D deficient. Using the 20ng/ml threshold, 57.2% of patients were classified as vitamin D deficient, whereas using the 30ng/ml threshold, the same was true for 85.5% of the patients.

### Statistical analysis

Statistical analysis was carried out using SPSS for Windows version 23 software package (Statistical Package for Social Sciences, IBM Corp. Armonk, NY, USA).

For continuous variables, we used independent sample t-test for equality of means to assess whether there were any statistically significant differences between the means of the outcome values. For categorical variables, Pearson’s chi-squared test and Fisher's exact test were used to assess differences in the frequencies between the groups. Nonparametric tests were used as indicated.

We considered differences with *p*-values of <0.05 as significant.

## Results

The mean age of the patients was 53.78 years (range 14–86) and 75% were female. Preoperatively, only 22.6% had a confirmed malignancy, whereas for the rest, the preoperative workup did not confirm TC. These patients underwent thyroidectomy for benign toxic or nontoxic multinodular goitre and a small percentage for Graves’ disease (3.1%). Final histology revealed malignancy in 56% of cases (group B = 183), mainly microcarcinomas (34.8%) and to a lesser extent macrocarcinomas (21.2%), whereas in 44% of patients final histology confirmed a benign disease (group A = 144). The mean concentration of plasma 25(OH)VitD level was 19.69ng/ml (sd 11.34) for group A and 19.82ng/ml (sd 9.59) for group B; there was no significant difference between the two groups (*p* = 0.917). The different parameters and comparisons between the two groups are given in Table 1.

**Table 1:** Comparison of patients with non-thyroid cancer vs thyroid cancer

	Non-thyroid cancer patients (*N* = 144)	Thyroid cancer patients (*N* = 183)	*p*-value
Age (years)	54.64 (15.09)	53.09 (13.68)	0.331
BMI (kg/m^2^)	27.11 (5.22)	26.92 (5.08)	0.270
Weight (kg)	77.24 (17.46)	74.38 (15.83)	0.149
Height (cm)	166.14 (8.42)	165.62 (8.83)	0.616
TSH (μIU/mL) ^ab^	1.19 (1.08)	1.55 (1.33)	**0.013**
Gland weight	51.51 (51.80)	32.11 (28.8)	**<0.001**
Anti-TGa	102.56 (323)	166.95 (691)	0.428
Anti-TPOa	156.52 (573)	174.37 (1026.73)	0.885
Antibodies+ (%)	27.1	32.7	0.231
Right A-P size (mm)	19.89 (10.16)	18.4 (9.09)	0.168
Left A-P size (mm)	19.94 (11.37)	16.54 (7.28)	**0.001**
Biggest nodule size (mm)	21.52 (15.32)	17.56 (13.58)	**0.014**
Preoperative Ca levels (ng/ml)	9.59 (0.49)	9.60 (0.52)	0.836
25(OH)VitD (ng/ml)	19.69 (11.34)	19.82 (9.59)	0.917
PTH (ng/dl)	56.20 (24.35)	61.80 (54.62)	0.429
Drinking (%)	7.0	10.6	0.198
Smoking (%)	40.6	38.8	0.420
Women (%)	72.2	77.2	0.184
25(OH)D < 10ng/ml (%)	16.7	14.2	0.322
25(OH)D < 20ng/ml (%)	59	56.3	0.350
25(OH)D < 30ng/ml (%)	88.9	83.6	0.114

^a^Nonparametric test was used to compare the variables.

Unless noted otherwise, values are given as mean (sd); ^b^median (interquartile range)

A-P = anterior-posterior; BMI = body mass index; 25(OH)D = 25-hydroxyvitamin D; PTH = parathormone; Tg = thyroglobulin; TPO = thyroid peroxidase; TSH = thyroid-stimulating hormone

When we compared the two groups according to the Endocrine Society classification (three levels of vitamin D: deficiency, inadequacy, adequacy), there was no statistically significant difference between them (*p* = 0.366). We further compared the two groups based on the three different threshold levels of vitamin D (Table 1).

To find any possible association with microcarcinomas, we further undertook a subgroup analysis including only microcarcinomas (group A, non-thyroid cancer [*N* = 144] vs group B, microcarcinomas [*N* = 112]). There was no statistically significant difference between the groups in all the aforementioned analyses (VitD <10ng/ml, *p* = 0.550; VitD <20ng/ml, *p* = 0.242; VitD <30ng/ml, *p* = 0.170; and for the three categories of vitamin D, *p* = 0.508). The same was true when we performed a subgroup analysis including only macrocarcinomas. Finally, we conducted a separate analysis to find out whether there were any differences in the variables between patients with vitamin D levels below (group deficient) and above (group adequate) 10ng/ml (Table 2).

**Table 2:** Comparison of variables among all vitamin D thresholds

	Vitamin D < 10ng/ml Patients (*N* = 51)^a^	Vitamin D > 10ng/ml Patients (*N* = 276)^a^	*p*-value
Thyroid cancer (%)	52	56.7	0.322
Smoking (%)	54.5	37.7	**0.027**
Age (years)	54.39 (15.77)	53.67 (14.00)	0.740
BMI (kg/m^2^)^b^	28.05 (6.35)	27.43 (5.84)	0.521
All other factors measured	NS	NS	>0.05
	Vitamin D < 20ng/ml Patients (*N* = 193)^a^	Vitamin D > 20ng/ml Patients (*N* = 134)^a^	
Thyroid cancer (%)	54.8	57.6	0.350
Age (years)	55.23 (15)	51.83 (13)	**0.032**
BMI (kg/m^2^)^b^	26.45 (4.68)	28.37 (6.62)	**0.006**
All other factors measured	NS	NS	>0.05
	Vitamin D < 30ng/ml Patients (*N* = 279)^a^	Vitamin D > 30ng/ml Patients (*N* = 48)^a^	
Thyroid cancer (%)	54.4	65.2	0.114
Age (years)	54.26 (14.7)	50.87 (13.45)	0.136
BMI (kg/m^2^)^b^	27.90 (5.9)	25.43 (5.24)	**0.01**
All other factors measured	NS	NS	>0.05

^a^All vitamin D thresholds

^b^Nonparametric test was used to compare the variables

Values are given as mean (sd) unless stated otherwise

NS = non significant

Apart from smoking, there were no significant differences in the continuous or in the categorical variables, including the presence or otherwise of malignancy (microcarcinomas or macrocarcinomas). There were more smokers in the vitamin D-deficient group, in line with data from the literature, supporting a reduced ability of sinus mucosa to activate circulating vitamin D levels.^10,11^ When we performed the analysis between patients with vitamin D levels below and above 20ng/ml, only body mass index (BMI) and age were found to be statistically different. This finding is a consistent association found in the published literature, with age and body fat content being inversely correlated to serum 25(OH)VitD concentrations, mainly because of reduced mobility and decreased sun exposure.^12^ Finally, after using a 25(OH)VitD level of 30ng/ml, the same was true only for BMI (Table 2).

## Discussion

Differentiated TC constitutes the most common malignancy of the endocrine system, with a progressively increasing trend in past years.^1,13,14^ This goes along with the steadily growing detection of thyroid nodules, resulting in an upward incidence of TC, mainly microcarcinomas. This is believed to happen because of improved and evolved imaging, better and more thorough pathological examination of specimens and more extensive screening of the population.^1,15^ Although significant efforts have been made to detect potential risk factors predisposing to TC, there are few well-established risk factors in the literature such as age, sex, BMI, income, radiation exposure and family history.^2,16–21^ Literature data have mainly shown a correlation between TC and high levels of TSH, although one study found that low levels of TSH could be a potential risk factor.^22–26,27^ Other studies report a positive correlation between autoimmune thyroid diseases (Hashimoto's thyroiditis, Graves’ disease) and TC.^28–30^

The role of 25(OH)VitD (the main circulating form of vitamin D in the human body) in carcinogenesis generally remains controversial, so it is extremely important to clarify this hypothesis taking into account the alarming worldwide rates of hypovitaminosis D and its possible consequences.^31^ According to a meta-analysis by Wang *et al* including 20 studies, there is a clear association of autoimmune thyroid disease (Hashimoto's thyroiditis and Graves’ disease) and decreased levels of 25(OH)VitD.^8^ Moreover, there are reports that support the association of vitamin D deficiency with other cancers. Epidemiological studies and even a systematic review of prospective studies supported the association of 25(OH)VitD levels with colon, breast and prostate cancer.^32–34^ Finally, a correlation between vitamin D deficiency and TC has been reported, although this remains controversial, considering papers in the literature supporting both sides.^24,35–42^ Two published meta-analyses from 2018 essentially suggest a possible correlation between vitamin D deficiency and thyroid malignancy.^2,3^ The first, carried out by Hu *et al* and including 11 studies, came to the conclusion that high levels of vitamin D may be related to a decreased risk for TC.^2^ The other, conducted by Zhao and coworkers, suggested that vitamin D deficiency may be a risk factor for TC, without being able to document a relationship with specific pathological characteristics in patients with thyroid malignancy.^3^

Comparing the results of our work with the two meta-analyses, we notice that they are in conflict. Our study found no significant difference in levels of serum 25(OH)VitD among patients who were operated for a benign thyroid lesion and those who had cancer, taking into consideration all possible accepted limits for ‘vitamin D deficiency’. This is in line with one of the basic studies used in the meta-analysis of Hu (weight 27.02%). In a study by Danilovic *et al*, no difference in the vitamin D levels between patients with TC and those with BTD was found (*p* = 0.153) and this was the conclusion stated.^39^ Moreover, in line with our results, in the study by Penna-Martinez *et al* included in the aforementioned meta-analysis (weight 17.55%), no significant difference in the levels of 25(OH)VitD was observed between patients with TC and healthy individuals.^38^ The only differences found were in 1,25(OH)D3 levels, but there was significant difference between the age of control and study population. Laney *et al* clearly found no difference in the levels of vitamin D between patients with TC and those without.^36^ Jonklaas *et al* reported no association between 25(OH)VitD and TC and only correlated high TSH with TC, as in our study.^37^ Moreover, Choi *et al*, conducting their study in a Korean population, suggested that elevated values of vitamin D may protect against TC, but no significant association was found in euthyroid patients without autoimmune thyroid disease.^24^ Overall, in the meta-analysis of Hu *et al*, only three of the nine included studies demonstrated an association between low vitamin D levels and TC, including their own work.^2,35,40^ 25(OH)VitD concentration as a continuous variable was recorded in only seven studies, and the result of the meta-analysis suggested that 25(OH)VitD levels tended to be lower in patients with TC than in controls. However, there is heterogeneity among the studies. Some include healthy individuals as control group, whereas other use patients with BTD, a fact that may constitute selection bias. In addition, two studies including cases with medullary TC may have affected the results.

Hu *et al*, in their case–control analysis (also included in their meta-analysis), compared patients with TC and healthy individuals not having a thyroid pathology, which may have influenced their findings.^2^ They surprisingly found that the mean plasma concentration of vitamin D was 22.09 (sd 6.22) for cancer patients and 23.94 (sd 6.38) for controls and that this difference was significant (*p* = 0.005). In the retrospective study by Roskies *et al*, the results were based on only 12 patients with low vitamin D vs 88 patients with vitamin D sufficiency with a wide range of odds ratios.^35^ Finally, in the study of Sahin *et al*, there were statistically significant differences in many covariates between controls and patients with cancer, all of which could have been confounding factors and affected the results.^40^ In contrast to this study but in line with most of the studies included in the meta-analysis, we found slightly lower 25(OH)VitD levels for the non-cancer group; however, these were statistically insignificant. The only differences we observed between the two groups were higher levels of serum TSH and a lower weight of the thyroid gland in the cancer group, also in line with other similar studies.^37,39^ TSH is the major growth factor for thyrocytes. Although its trophic growth effects through TSH receptors on tumour cells are well established, its pathogenic role in TC is mainly unknown.^23^

Furthermore, when we performed a subgroup analysis including only microcarcinomas at first and then macrocarcinomas, the results were similar.

The second meta-analysis of Zhao *et al* published in the same year included 14 studies. Three of the main studies included in the previous meta-analysis of Hu *et al* were not included in this one. Nine extra studies were included in this meta-analysis, so by definition, there are data missing from both papers (publication bias). In this study Zhao *et al* performed three separate analyses according to when was vitamin D measured.^3^ Vitamin D was tested before surgery in four studies, in two of which lower levels of vitamin D were found in patients with TC than in controls.^43,44^ When vitamin D was measured after the surgical procedure, the analysis showed no difference in serum 25(OH)VitD levels between the two groups. Furthermore, only five studies included in the meta-analysis measured the relative risk for TC due to vitamin D deficiency.^24,35,36,38,44^ Although a 30% increased risk for malignancy was mentioned, only Roskies *et al* found a statistically significant difference, but there are limitations in their study as discussed earlier.^35^ Moreover, two studies conducted in a Chinese population came to completely different conclusions.^45,46^ Jiang *et al* showed that patients with TC had lower levels of 25(OH)VitD compared with healthy controls, whereas Ye and coworkers reported exactly the opposite.

### Study limitations

We recognise that our study has several weaknesses. First, the main limitation is a lack of adjustment for some possible confounding factors such as age and BMI. The lack of adjusted TSH levels may be an important limitation taking into consideration its possible predisposing role in formation of TC. The second weakness of our study is that there is no subgroup analysis on different types of thyroid tumours. Third, there was no seasonal measurement of 25(OH)VitD values. The biosamples were taken throughout the year and from different laboratories. Nevertheless, all measurements were collected just before surgery, and not after or several months before the surgical procedure, which we believe is a strength of our work. Moreover, despite the fact that this is a single institution observational study, it is based on a prospectively collected database, one of the largest in the literature, including a relatively sufficient analysed population. Larger-scale multi-institutional studies that may confirm our findings are needed.

In conclusion, our study found no significant association between low vitamin D levels and TC, comparing two groups of patients with well-differentiated TC and BTD, even when we used all possible accepted limits for ‘vitamin D deficiency’. We strongly believe that other confounding factors may have led to the results of the meta-analyses and other studies presenting similar findings. Nevertheless, there are reports of association of low vitamin D levels with TC, and mainly with autoimmune thyroid diseases, when compared with healthy individuals. We believe that a correlation of low vitamin D and thyroid disorders may exist, given the possible connection between TC and autoimmunity. However, there is clearly no consensus on the association of low vitamin D levels with TC, and further epidemiological studies, accounting for all possible confounding factors, are necessary to confirm or reject this hypothesis.

## Author contributions

All authors contributed equally to the design, statistical analysis, writing and editing of the study.
